# Assessment of the anticancer and antimetastatic effects of monocarbonyl analogs of curcumin, C66 and B2BrBC, in breast cancer cells

**DOI:** 10.1186/s12935-026-04184-8

**Published:** 2026-01-21

**Authors:** Radoslav Stojchevski, Sara Velichkovikj, Jane Bogdanov, Katerina Dragarska, Ivana Todorovska, Nikola Hadzi-Petrushev, Mitko Mladenov, Leonid Poretsky, Dimiter Avtanski

**Affiliations:** 1 Friedman Diabetes Institute, Lenox Hill Hospital, Northwell Health, 110 E 59th Street, Suite 8B, Room 837, New York, 10022 NY USA; 2 Institute of Molecular Medicine, The Feinstein Institutes for Medical Research, Manhasset, NY USA; 3 Donald and Barbara Zucker School of Medicine at Hofstra/Northwell, Hempstead, NY USA; 4 Office of Clinical Research, Lenox Hill Hospital, Northwell Health, New York, NY USA; 5 Faculty of Natural Sciences and Mathematics, Institute of Chemistry, Ss. Cyril and Methodius University, Skopje, Macedonia; 6 Faculty of Natural Sciences and Mathematics, Institute of Biology, Ss. Cyril and Methodius University, Skopje, Macedonia; 7 Institute of Bioelectronic Medicine, The Feinstein Institutes for Medical Research, Manhasset, USA

**Keywords:** Curcumin, Curcumin analogs, Monocarbonyl analogs of curcumin (MACs), Antioxidant activity, Anticancer properties, Breast cancer, Epithelial-to-mesenchymal transition (EMT)

## Abstract

**Background:**

Curcumin, a natural compound found in turmeric (*Curcuma longa*), demonstrates anticancer properties; however, it is characterized by poor bioavailability and stability. This study investigates the stability, antioxidant activity, and anticancer effects of two monocarbonyl analogs of curcumin (MACs), C66 and B2BrBC, in in vitro breast cancer models.

**Methods:**

Stability and antioxidant activity of C66 and B2BrBC were assessed using spectrophotometric assays. Their effects on breast cancer cells (MCF-7, BT-474, MDA-MB-231) were evaluated through MTT assay, wound-healing assay, and caspase-3 fluorescence microscopy. EMT modulation was examined via RT-qPCR, Western blot, and immunofluorescence analyses. A MILLIPLEX protein assay was used to analyze cancer metastasis-related protein expression.

**Results:**

C66 and B2BrBC demonstrated enhanced stability compared to curcumin. Both compounds significantly reduced breast cancer cell viability and migration, with B2BrBC showing higher potency. They effectively suppressed EMT, reversing EMT-inducer effects on epithelial and mesenchymal markers. C66 and B2BrBC modulated the expression of several metastasis-related proteins, including DKK1, OPG, and GDF15, in a cell line-dependent manner.

**Conclusions:**

C66 and B2BrBC exhibit improved stability and potent anticancer effects in breast cancer cells, effectively inhibiting cell viability, migration, and EMT. These compounds show promise as potential therapeutic agents for breast cancer, warranting further investigation in in vivo models.

**Supplementary Information:**

The online version contains supplementary material available at 10.1186/s12935-026-04184-8.

## Background

Curcumin (CUR) is a natural polyphenolic compound in the rhizome of turmeric (*Curcuma longa L.*) used for centuries in culinary and traditional medicine in treating cardiovascular, pulmonary, digestive, renal, and nervous conditions [1–5]. In addition, CUR displays anticancer effects in various cancer types, such as lung, colon, stomach, pancreatic, cervical, prostate, and breast [6–8]. These effects are mainly attributed to CUR’s strong anti-inflammatory and antioxidant properties [9]. However, although promising, the use of CUR as a therapeutic agent is limited due to its poor absorption and bioavailability [10]. These drawbacks have been addressed by using various approaches, including CUR inclusion in adjuvants, liposomes, or nanoparticles [11]. Structural modification of the CUR molecule to improve its solubility and absorption is another promising strategy. Monocarbonyl analogs of curcumin (MACs) without the β-diketone moiety have been shown to have enhanced stability in vitro and improved pharmacokinetic profile in vivo [12].

One key area where CUR’s or MACs’ antioxidant effects may play a significant role is in mitigating oxidative stress, which is closely linked to the epithelial-to-mesenchymal transition (EMT), a critical step in cancer progression and metastasis [13–15]. The mechanism of this connection involves the activation of TGFβ/Smad and Wnt/β-catenin signaling pathways that are crucial for EMT reprogramming [16]. Reactive oxygen species (ROS) can affect TGFβ, which in turn can either protect from or contribute to cellular neoplastic transformation [17], as it is suggested that EMT may provide a survival benefit for cells undergoing oxidative stress [18].

The aim of the present study was to evaluate the stability, antioxidant activity, and potential anticancer effects in vitro of two MACs, 2,6-bis(2-trifluoromethylbenzylidene)cyclohexanone (C66) and 2,6-bis(2-bromobenzylidene)cyclohexanone (B2BrBC), using various breast cancer cell lines. Specifically, we sought to (1) assess the stability and biocompatibility of C66 and B2BrBC compared to CUR, (2) investigate their effects on breast cancer cell viability, apoptosis, and migration, (3) examine their impact on EMT through analysis of key markers and signaling pathways, and (4) evaluate their influence on the expression of proteins involved in cancer metastasis. By examining these effects, we aimed to gain a better understanding of the potential of these structural analogs as therapeutic agents for breast cancer, addressing the limitations of CUR.

## Materials and methods

### Materials

CUR was purchased from Millipore Sigma (Cat. # C7727). (2*E*,6*E*)−2,6-bis[(2-trifluoromethyl)benzylidene]cyclohexanone (C22H16F6O, C66) and (2*E*,6*E*)−2,6-bis(2-bromobenzylidene)cyclohexanone (C20H16Br2O, B2BrBC) were synthesized via cross-aldol condensation, adapted from previously published methods [19–21]. Breast cancer (MCF-7, MDA-MB-231, and BT-474) and non-carcinogenic breast epithelial (MCF-10 A) cells were purchased from the American Type Culture Collection (ATCC) (Cat. # HTB-22, HTB-26, HTB-20, and CRL-10317, respectively).

### Cell cultures

MCF-7 and MDA-MB-231 cells were grown in DMEM/F12 (50:50) medium (Corning, Cat. # 10–092-CM), BT-474 cells in RPMI1-1640 medium (ATCC, Cat. # 30–2001), and MCF-10 A cells in Mammary Epithelial Cell Basal medium (ATCC, Cat. # PCS-600-030) supplemented with Mammary Epithelial Cell Growth kit (ATCC, Cat. # PCS-600-040), 10% FBS (Avantor, Cat. # 89510-186) and antibiotic/antimycotic solution (Corning, Cat. # 30-004-Cl) and incubated at 37 °C and 5% CO_2_.

### Stability assay

C66, B2BrBC, and CUR were dissolved in DMSO at a concentration of 1 mg/ml. The stocks were then diluted with PBS buffer (10 mM sodium phosphate, 150 mM NaCl, pH 7.4) to achieve a final concentration of 20 µM. Absorption spectra were recorded from 250 to 600 nm using a UV − Vis spectrophotometer (Cary 60 UV-Vis spectrophotometer, Agilent) for a span of 25 min at 5 min intervals.

### Hemolytic assay

The assay was performed following the method described by Kumar et al. [22]. Blood was drawn from the rat’s abdominal aorta in the presence of K_3_EDTA as an anticoagulant. The blood was centrifuged at 1,500 RPM at 4 °C for 10 min to isolate the red blood cells (RBCs). The RBCs were washed three times and resuspended to 4% v/v in PBS buffer (35 mM sodium phosphate, 150 mM NaCl, pH 7.4). Next, 100 µL of RBC suspension was mixed with 100 µL C66, B2BrBC, or CUR of each concentration in the range of 25–300 µM. The mixtures were incubated for 2 h at 37 °C. RBC suspension in PBS was used as a negative control, and RBCs incubated with 0.1% Tween-20 represented a positive control for hemolysis. After the incubation period, the tubes were centrifuged, and 20 µL of supernatant from each tube was added to 80 µL of PBS buffer in a 96-well microtiter plate. The absorbance due to the presence of hemoglobin in each well was measured at 415 nm. Percentage hemolysis was calculated using the following equation: $$\%\:hemolysis$$ $$=\frac{OD\:of\:sampple-OD\:of\:negative\:control}{OD\:of\:positive\:control-OD\:of\:negative\:control}$$$$ \; x\:100$$. Three independent experiments were used to get the final hemolytic percentage.  

### DPPH radical scavenging assay

Free radical scavenging activity of C66, B2BrBC, and CUR was tested by DPPH radical scavenging assay as described by Bankova et al. [23]. First, an ethanolic solution of DPPH was prepared by dissolving 1.25 mg DPPH in 10 ml 96% ethanol to obtain 317 µM DPPH solution. The solution was diluted twice before use. Reaction mixtures containing 10 or 20 µL of different concentrations of MACs dissolved in DMSO were added to 190 or 180 µL of DPPH ethanolic solution in a 96-well microtiter plate and incubated at 25 °C for 30 min. The absorbances of the wells containing a series of dilutions of MACs (with final concentration range as follows: CUR 1–50 µg/ml; B2BrBC and C66 1,000–2,000 µg/ml) were measured at 535 nm on a microplate spectrophotometer. Sample blanks were prepared using 96% ethanol instead of DPPH ethanolic solution. As a control for this analysis, 180 or 190 µL of DPPH ethanolic solution was mixed with 10 or 20 µL of DMSO. The percentage of DPPH radical scavenging activity (RSA%) was then calculated using the following equation: $\:RSA\%=100-\frac{\left(\mathrm{A}\mathrm{s}-\mathrm{A}\mathrm{b}\right)\:\mathrm{x}\:100}{Ac}$, where *As* is the absorbance of the sample, *Ab* is the absorbance of the respective sample blank, and *Ac* is the absorbance of the control. Ascorbic acid was used as a positive control.

### Catalase and superoxide dismutase colorimetric assays

Protein fraction was extracted from MCF-7, MDA-MB-231, and BT-474 cells that underwent treatment with C66 or B2BrBC (100 µM) using radioimmunoprecipitation assay (RIPA) lysis buffer (Thermo Fisher Scientific, Cat. # 89901) supplemented with a Protease & Phosphatase Inhibitor Cocktail (Thermo Fisher Scientific, Cat. # 78440). The samples were centrifuged to remove cell debris, and protein concentrations were quantified using BCA Protein Assay (Thermo Fisher Scientific, Cat. # 23225). Catalase Assay Kit (Cayman Chemical, Cat. # 707002) and Superoxide Dismutase Assay Kit (Cayman Chemical, Cat. # 706002) were used to assess antioxidant enzyme activities following the manufacturer’s protocols.

### MTT assay

MCF-7, MDA-MB-231, BT-474, and MCF-10 A cells were seeded at a density of 2.5 × 10^4^ cells/well in a 96-well plate and incubated for 24 h at 37 °C and 5% CO_2_, after which the cell culture medium was replaced with serum-free medium and incubated for an additional 16–18 h. After this starvation period, the cells were treated with C66 or B2BrBC dissolved in DMSO to a final concentration of 100 µM for a duration of 24 h. DMSO-treated cells were used as a control. After the treatment, the medium was removed, an MTT reagent (Abcam, Cat. # ab211091) was added, and the cells were incubated for 3 h. The insoluble formazan crystals were dissolved using a solubilization solution and quantified by measuring the absorbance at 562 nm on a microplate reader (BioTek elx800). Experiments were repeated five times in triplicate. The data were presented as a % of viability compared to the control.

### Caspase-3 dye immunofluorescence microscopy

MCF-7, MDA-MB-231, and BT-474 were seeded at a density of 1.5 × 10^5^ cells/well in a 24-well plate in a complete medium and treated with C66 or B2BrBC dissolved in DMSO to a final concentration of 100 µM. Cells treated with DMSO were used as controls. After 24 h, the conditioned medium was replaced with fresh medium containing 5 µM of the Green Caspase-3 Dye (Millipore Sigma, Cat. # SCT101). The cells were incubated at room temperature for 1 h and directly observed under a fluorescence microscope using 20x magnification (EVOS FL AUTO, Applied Biosciences). Experiments were repeated three times, and data were presented as a number of caspase-3-positive cells in each condition.

### Wound-healing assay

MCF-7, MDA-MB-231, and BT-474 cells were seeded at a density of 4 × 10^4^ cells/well in 24-well cell culture plates with silicone inserts (Culture-Insert 2 Well 24, Ibidi, Germany, Cat. # 80242) and incubated at 37 °C and 5% CO_2_ until a confluent monolayer was formed. Then, the silicon insert was removed, the cells were washed once with PBS, and the medium was replaced with fresh complete medium supplemented with C66 or B2BrBC, each at 100 µM, or DMSO (control). Series of images were taken at 10x magnification using EVOS XL Core Imaging System (Thermo Fisher Scientific) in the same areas at four time-points: 0, 24, 72, and 120 h (MCF-7); 0, 18, 36, and 54 h (BT-474); and 0, 18, 24, and 48 h (MDA-MB-231). Experiments were repeated five times in triplicate. For every image, gap width was measured in three different areas, and the data for each condition were presented as a % difference in scratch width for each time point compared to their initial width at 0 h.

### Transwell migration assay

MCF-7, MDA-MB-231, and BT-474 cells were serum-starved for 24 h in their respective media before the assay. Cells were seeded at a density of 1 × 10⁵ cells/mL into 24-well cell migration plate inserts with an 8-µm pore size polycarbonate membrane (QCM™ 24-Well Colorimetric Cell Migration Assay, Millipore, USA, Cat. # ECM508). The inserts were placed over complete media with 10% FBS as a chemoattractant in the lower chambers, containing C66 or B2BrBc at a final concentration of 100 µM, with DMSO control wells included as vehicle controls. After incubation for 24 h at 37 °C in a 5% CO₂ humidified atmosphere, migrated cells on the lower membrane surface were fixed with 3.7% formaldehyde and stained using the kit-provided Cell Stain Solution. Images were captured at 10× magnification using the EVOS XL Core Imaging System (Thermo Fisher Scientific). The stained dye was then solubilized with Extraction Buffer, and absorbance was measured at 562 nm using a microplate reader (BioTek elx800), with experiments repeated three times and data presented as a % of migration compared to control.

### RT-qPCR analyses

For RT-qPCR analyses, 0.3 × 10^6^ MCF-7 cells/well were seeded in 6-well plates in a complete medium. EMT-inducing medium supplement (Bio-techne, Cat. # CCM017) containing TGFβ1, Wnt5a, anti-E-cadherin, anti-sFRP1, and anti-Dkk-1 antibodies was also added to the cells at the time of seeding. The cells were incubated at 37 °C with 5% CO_2_ for 48 h. After incubation, the medium was replaced with a fresh, complete medium. A new EMT supplement, along with C66 or B2BrBC (each at 100 µM) or DMSO (control), was added to the cells, and the incubation was continued for an additional 72 h. Total RNA was extracted using TRIzol reagent (Thermo Fisher Scientific, Cat. # 15596026) and quantified using a NanoDrop One spectrophotometer (Thermo Fisher Scientific). Samples were normalized to 1 µg total RNA, then converted to cDNA using qScript cDNA SuperMix (Quantabio, Cat. # 95048) and SimpliAmp Thermal Cycle (Applied Biosystems, ThermoFisher Scientific). Gene expression was quantified using PerfeCTa SYBR Green FastMix (Quantabio, Cat. # 95072) and QuantStudio 3 Real-Time PCR System (Applied Biosystems, Thermo Fisher Scientific) using the following conditions: 2 min/50°C, 10 min/95°C, 15 s/95°C and 1 min/60°C (40 cycles), 15 s/95°C, 1 min/60°C, 15 s/95°C. The primer sequences and their characteristics are listed in Supplementary Table 1 [24–27]. The obtained threshold cycle (C*t*) values were analyzed with QuantStudio Design & Analysis Software using the delta-delta C*t* method (ΔΔC*t*) and GAPDH as a housekeeping gene. Results were obtained from four independent experiments, and data were presented as fold-change relative to vehicle control.

Additionally, RT-qPCR array analysis was performed using the Human Epithelial to Mesenchymal Transition kit (Qiagen, Cat. # 330231 PAHS-090ZA). The genes included in the panel and their functions are listed in Supplementary Tables 2 and Supplementary Table 3. Data analysis was performed using QIAGEN web-based software GeneGlobe DataAnalysis Center (https://geneglobe.qiagen.com/us/analyze) by using the geometric mean of the C*t* values of the genes of interest against the housekeeping genes: *ACTB*, *B2M*, *GAPDH*, *HPRT1*, and *RPLP0*. Results were obtained from three independent experiments. The differences in gene expression between the treatment and control groups were quantified as a fold-change through the ΔΔC*t* method.

### Western blot analyses

For western blot analyses, MCF-7 cells were seeded in 100 mm cell culture dishes at a density of 2.2 × 10^6^ cells/well and underwent treatment with C66 or B2BrBC (each at 100 µM) after EMT induction. Total cellular protein was extracted with RIPA buffer (Thermo Fisher Scientific, Cat. # 89901) supplemented with a protease and phosphatase inhibitor cocktail (Thermo Fisher Scientific, Cat. # 78440). Samples were centrifuged to remove cell debris, and protein concentrations were quantified using BCA Protein Assay (Thermo Fisher Scientific, Cat. # 23225); then, all samples were normalized to 10 µg total protein. Proteins were separated by SDS-PAGE using 4–12% gradient gels (Thermo Fisher Scientific, Cat. # NW04120BOX) and transferred onto 0.2 μm nitrocellulose membranes (GE Healthcare, Cat. # 10600015). Non-specific binding was blocked by overnight incubation at 4 °C with 5% non-fat dry milk. Membranes were blotted with primary antibodies overnight at 4 °C (Supplementary Table 4). The next day, membranes were incubated for 1 h at room temperature with corresponding anti-rabbit or anti-mouse HRP-conjugated secondary antibodies (Supplementary Table 4). Immunoreactive protein bands were visualized using SuperSignal West Pico Chemiluminescent Substrate (Cell Signaling Technology, Cat. # 34580) and MyECL imager (Thermo Fisher Scientific). Densitometry analyses were carried out using ImageJ software [28].

### Immunofluorescence microscopy

MCF-7 cells were seeded at a density of 1 × 10^4^ cells/well in 8-well chamber slides (Nunc Lab-Tek II, Thermo Fisher Scientific, Cat. # 154453), followed by treatment with C66 and B2BrBC (each at 100 µM) for 24 h. Slides were fixed with 4% formaldehyde for 15 min at room temperature and incubated in normal goat serum for 60 min to block the non-specific binding. Then, slides were incubated with β-Catenin (D10A8) primary antibody (Cell Signaling Technologies, Cat. # 8480) (1:100 dilution with antibody dilution buffer prepared with 0.1 g BSA, 30 µL Triton X-100, and 10 mL PBS), overnight at 4 °C, followed by fluorochrome-conjugated goat anti-rabbit secondary antibody (Cell Signaling Technologies, Cat. # 4412) (1:500 dilution in antibody dilution buffer) at room temperature for 1–2 h protected from light. Slides were mounted using Prolong Gold Antifade Reagent with DAPI (Cell Signaling Technologies, Cat. # 8961). Cells were imaged at 100x magnification using EVOS FL Auto Imaging System (Thermo Fisher Scientific) using DAPI and GFP filters and a combination of the two as a merged picture.

### MILLIPLEX array

MCF-7 and BT-474 cells (0.3 × 10^6^ cells/well) were seeded in 6-well plates with a complete medium and incubated for 24 h at 37 °C and 5% CO_2_. Then, the cell culture medium was replaced with a serum-free medium, and cells were incubated for an additional 16–18 h, followed by treatment with C66, B2BrBC (each at 100 µM), or DMSO (control) for an additional 24 h. After the treatment, 1 ml of the cell culture medium was collected, centrifuged, and stored at −80 °C until further analysis. MILLIPLEX array was performed using the Human Cancer/Metastasis Biomarker Magnetic Bead Panel–Cancer Multiplex Assay kit (MilliporeSigma, Cat. # HCMBMAG-22 K) following the manufacturer’s protocol. Data were calculated using xPONENT software for Magpix, version 4.2 (Luminex Corp.), and presented as a fold-change compared to the control.

### ADME analysis

CUR, C66, and B2BrBc structures were converted to simplified molecular input line entry system (SMILES) notation and analyzed using the SwissADME tool [29] to predict various absorption, distribution, metabolism, and excretion (ADME) properties, pharmacokinetic descriptors, and to estimate their drug-likeness. Key physicochemical descriptors, such as molecular weight, number of rotatable bonds, H-bond acceptors and donors, and topological polar surface area (TPSA), were evaluated to determine their compliance with drug-likeness criteria such as Lipinski’s and Veber’s rules. Additionally, parameters indicating water solubility, lipophilicity, and polarity were evaluated through Ali’s LogS, IlogP, and TPSA. Pharmacokinetic features, including passive human intestinal absorption (HIA), blood-brain barrier (BBB) permeation, and P-gp substrate prediction, were modeled using the BOILED-Egg model, distinguishing between physicochemical spaces conducive to BBB permeation and HIA absorption. Furthermore, the potential inhibitory effects of CUR, C66, and B2BrBC on five major isoforms of cytochrome P450 (CYP1A2, CYP2C19, CYP2C9, CYP2D6, CYP3A4) were assessed.

### Statistical analyses

GraphPad Prism 9.0 statistical software was used to process the results using One-way Analysis of Variance (ANOVA). Tukey’s post hoc test was used to determine the differences between group pairs, and they were considered statistically significant if *p* < 0.05. Student’s t-test was used to assess the differences between the groups from the RT-qPCR array, with significance defined as a p-value of < 0.05.

## Results

### Stability

**Fig. 1:**
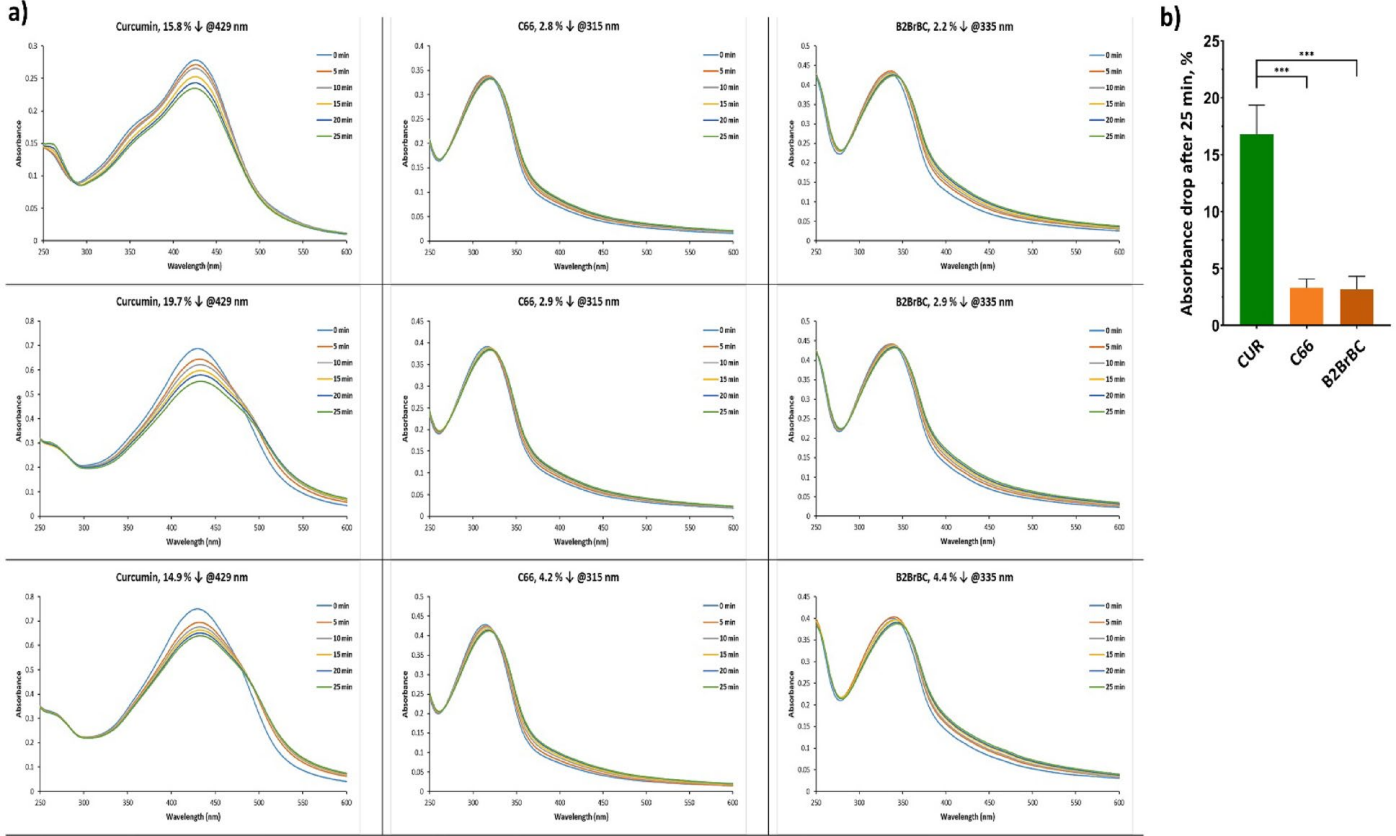
Stability of C66, B2BrBC, and CUR. To prepare stock solutions, CUR and MACs were dissolved in DMSO at a concentration of 1 mg/ml. The stocks were then diluted with PBS buffer (10 mM sodium phosphate, 150 mM NaCl, pH 7.4) to achieve a final concentration of 20 µM. (**a**) Absorption spectra were recorded from 250 to 600 nm using a UV − Vis spectrophotometer for a span of 25 min at 5 min intervals. (**b**) Data is presented as % of absorbance drop after 25 min ± SD. ***, *p* < 0.001(**a**)

Initially, we evaluated the stability of C66 and B2BrBC compared to CUR in simulated physiological conditions (Fig. 1). The results demonstrated a significant difference between the decrease in the maximum absorbance of CUR and the decrease in the maximum absorbance for C66 and B2BrBC after 25 min of the analysis. Namely, after 25 min, approximately 17% of the initially present CUR disintegrated, while the loss of the MACs was only about 3%, which confirmed the difference in their stability in physiological conditions. The difference in stability between C66 and B2BrBC solutions was found to be insignificant.

### Hemolytic assay

**Fig. 2:**
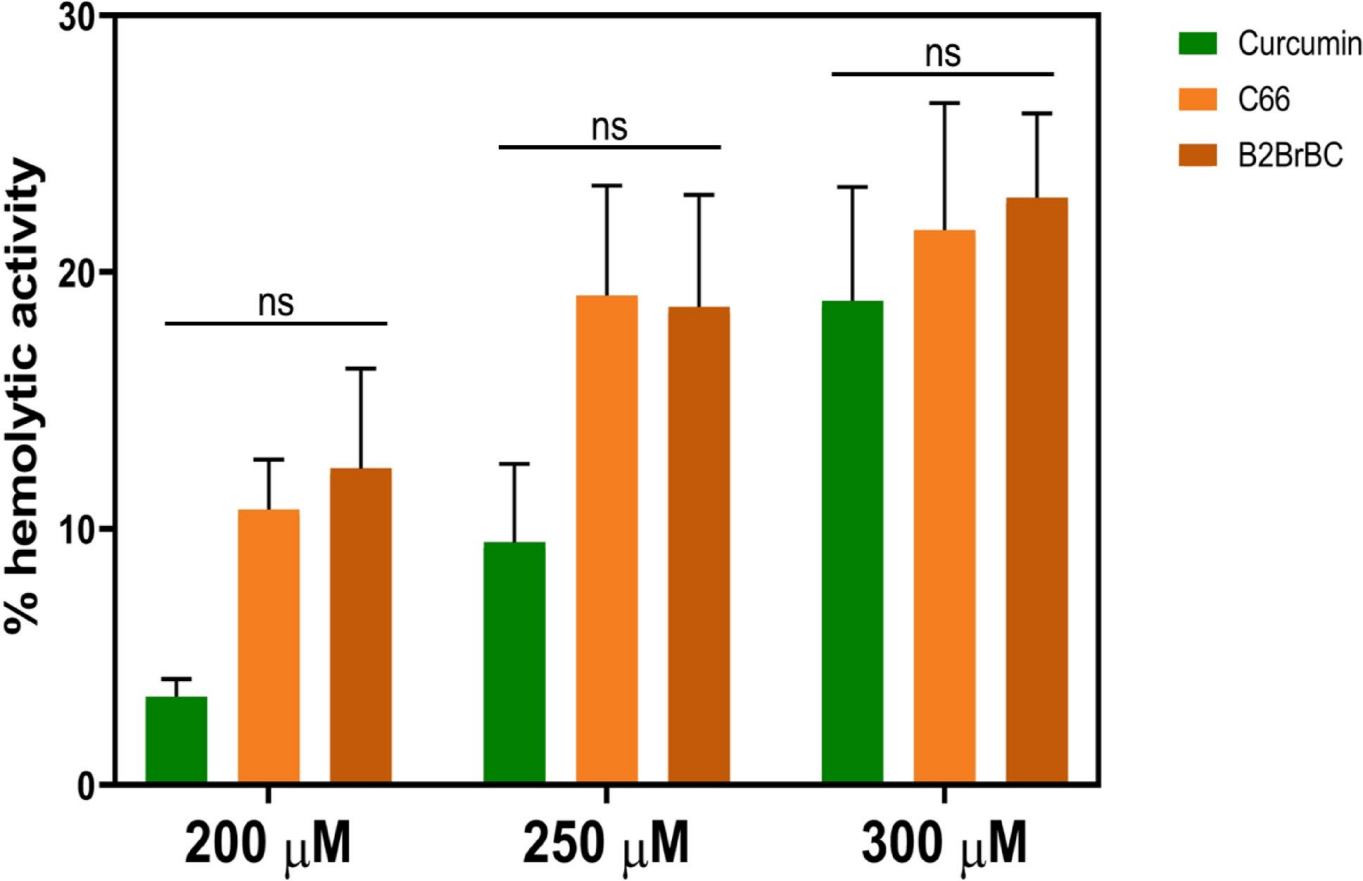
Hemolytic activity of C66, B2BrBC, and CUR. A hemolytic assay using rat red blood cells was used to determine C66 and B2BrBC biocompatibility compared to CUR at different concentrations (200, 250, and 300 µM). The absorbance due to the presence of hemoglobin in each well was measured at 415 nm and presented as a percentage of hemolysis ± SD. ns – nonsignificant

We further performed a hemolytic assay using rat red blood cells (RBCs) to determine C66 and B2BrBC biocompatibility (Fig. 2). Comparing the effects of C66 and B2BrBC to CUR at different concentrations (200, 250, and 300 µM), the results showed a dose-dependent increase in hemolysis, with the highest rate of ~ 20% observed at 300 µM. The analogs exhibited hemolytic activity that was not significantly different compared to CUR at all concentrations tested.

### Antioxidative effects of C66 and B2BrBC

**Fig. 3:**
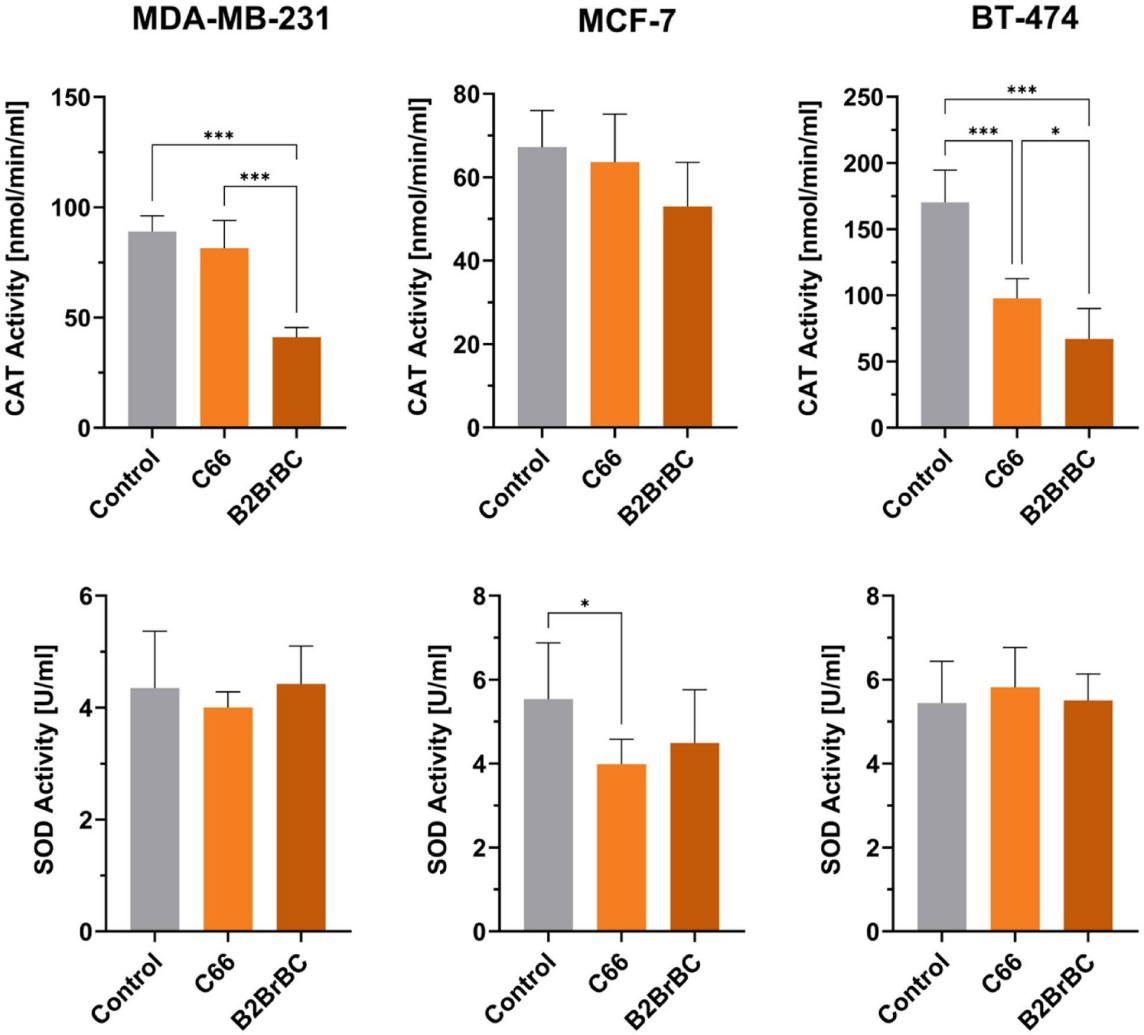
Antioxidant capacity of C66 and B2BrBC. A colorimetric assay was used to measure the activity of antioxidant enzymes catalase (CAT) and superoxide dismutase (SOD) in MCF-7, MDA-MB-231, and BT-474 cells in response to the treatment of C66 and B2BrBC. CAT activity is presented as nmol/min/ml, or the amount of enzyme that causes the formation of 1 nmol of formaldehyde per minute. SOD activity is presented as U/ml, or the amount of enzyme needed to exhibit 50% dismutation of the superoxide radical measured in change in absorbance per minute ± SD. *, *p* < 0.05; ***, *p* < 0.001

To evaluate the free-radical scavenging activity of C66 and B2BrBC compared to CUR, we performed a DPPH free-radical scavenging assay and used ascorbic acid as a control. Ascorbic acid exhibited a strong concentration-dependent increase in scavenging activity, reaching ~ 50% at approximately 49.2 µM (46.831–52.247 95% CI, EC_50_ value). CUR also showed a concentration-dependent increase in scavenging activity, but it is less potent than ascorbic acid, with an EC_50_ value of 84.7 µM (77.813–91.818 95% CI). The scavenging activity of C66 and B2BrBC was not detectable up to the highest tested concentration of 4,837 µM (2,000 µg/ml) and 4,629 µM (2,000 µg/ml), respectively (Supplementary Table 5). Additionally, we conducted colorimetric assays to measure the activity of antioxidant enzymes catalase (CAT) and superoxide dismutase (SOD) in MCF-7, MDA-MB-231, and BT-474 cells in response to the treatment of C66 or B2BrBC, each at 100 µM (Fig. 3). The results showed a significant decrease in CAT activity in B2BrBC-treated MDA-MB-231 and BT-474 cells, as well as C66-treated BT-474 cells. A reduction in the activity of SOD was detected in C66-treated MCF-7 cells.

### In vitro effects of C66 and B2BrBC on cell viability and motility in breast cancer cells

**Fig. 4:**
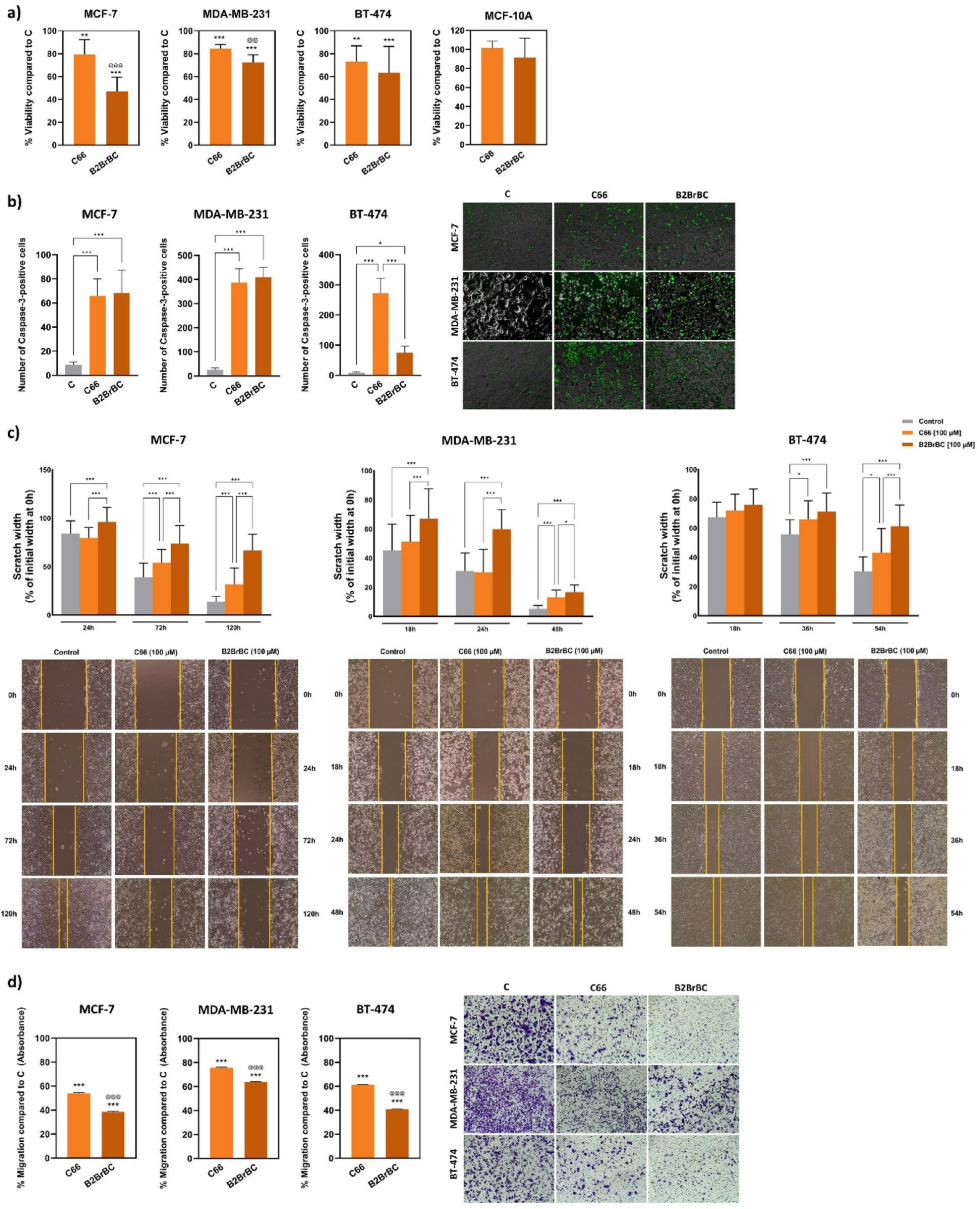
Effect of C66 and B2BrBC on breast cancer cell viability and migration. (**a**) MCF-7, BT-474, and MDA-MB-231 breast cancer cells and MCF-10 A non-carcinogenic breast epithelial cells were subjected to C66 or B2BrBC treatment (100 µM) for 24 h, and cell viability was measured using an MTT assay. Data are presented as % viability of C66- or B2BrBC-treated, compared to control (DMSO-treated) cells ± SD. **, *p* < 0.01; ***, *p* < 0.001 compared to control; ^@@^, *p* < 0.01; ^@@@^, *p* < 0.001 C66 vs. B2BrBC. (**b**) The level of apoptosis in MCF-7, BT-474, and MDA-MB-231 after treatment with 100 µM of C66 or B2BrBC for 24 h was evaluated by Caspase-3 dye fluorescence microscopy. The data were presented as a number of caspase-3 positive cells in each condition ± SD. *, *p* < 0.05; ***, *p* < 0.001. (**c**) Changes in the migration potency of MCF-7, BT-474, and MDA-MB-231 after treatment with 100 µM of C66 or B2BrBC for 24 h were assessed via the wound-healing assay. Phase-contrast microscope images of the wound openings were taken at 24, 72, and 120 h (MCF7 cells); 18, 36, and 54 h (BT-474); 18, 24, and 48 h (MDA-MB-231 cells) after the treatment. Cell migration was evaluated by measuring the gap width at three different locations in every picture. The data were presented as a % difference in gap width for each time point compared to their initial width at 0 h ± SD. *, *p* < 0.05; ***, *p* < 0.001. (**d**) Migration potency of MCF-7, BT-474, and MDA-MB-231 cells after treatment with 100 µM C66 or B2BrBc for 24 h was assessed via a Transwell migration assay. Cells were seeded in serum-free media over complete media with 10% FBS as a chemoattractant in the lower chambers, containing C66, B2BrBc, or DMSO (control). Brightfield images of migrated cells on the lower membrane surface were captured at 10× magnification, and cell migration was quantified colorimetrically, with data presented as a % of migration compared to DMSO control ± SD. ***, *p* < 0.001 compared to control; ^@@@^, *p* < 0.001 C66 vs. B2BrBC

To evaluate whether C66 and B2BrBC modulate cell viability in vitro, we performed a series of experiments using breast cancer MCF-7, MDA-MB-231, BT-474, and breast non-carcinogenic MCF-10 A cells. Cell viability was assessed by MTT assay after 24 h of treatment with C66 or B2BrBC (each at 100 µM) and compared to vehicle (DMSO)-treated cells. As demonstrated in Fig. 4a, C66 and B2BrBC significantly inhibited the viability of all three breast cell lines by 50–80%, with B2BrBC being more effective than C66. On the contrary, the treatment of MCF-10 A cells with these compounds did not significantly affect their cell viability. Fluorescence microscopy revealed that B2BrBC triggered higher caspase-3 activation in MCF-7 and MDA-MB-231 cells, while C66 induced higher activation in BT-474 cells (Fig. 4b). In these experiments, MDA-MB-231 cells showed noticeably the highest level of apoptosis after C66 or B2BrBC treatment.

To investigate the effect of C66 and B2BrBC on cell motility, we performed a wound-healing assay using MCF-7, BT-474, and MDA-MB-231 cells (Fig. 4c). The cells were treated with 100 µM of C66 or B2BrBC, and the wound width was measured at specific time points for each cell line. This experiment revealed significant migration inhibition in all the examined breast cancer cell lines following treatment with C66 or B2BrBC, with B2BrBC demonstrating much higher potency than C66 at each time point. To further validate these findings, we conducted a Transwell migration assay, which was consistent with the wound-healing assay, confirming significant migration inhibition across all cell lines, with B2BrBC exhibiting greater potency than C66 (Fig. 4d).

### Effect of C66 and B2BrBC on EMT of breast cancer cells

We further examined the effects of C66 and B2BrBC on EMT. We measured the expression of various epithelial (*E-cadherin* and *CK-18*) and mesenchymal (*fibronectin* and *vimentin*) genes, as well as factors regulating mesenchymal genes transcription (*snail* and *slug*) in MCF-7 cells using RT-qPCR analysis. EMT in MCF-7 was induced with a cocktail of EMT-inducing agents for a duration of five days. Subsequently, the cells were treated with 100 µM of C66 or B2BrBC for 72 h within the five-day period.

**Fig. 5:**
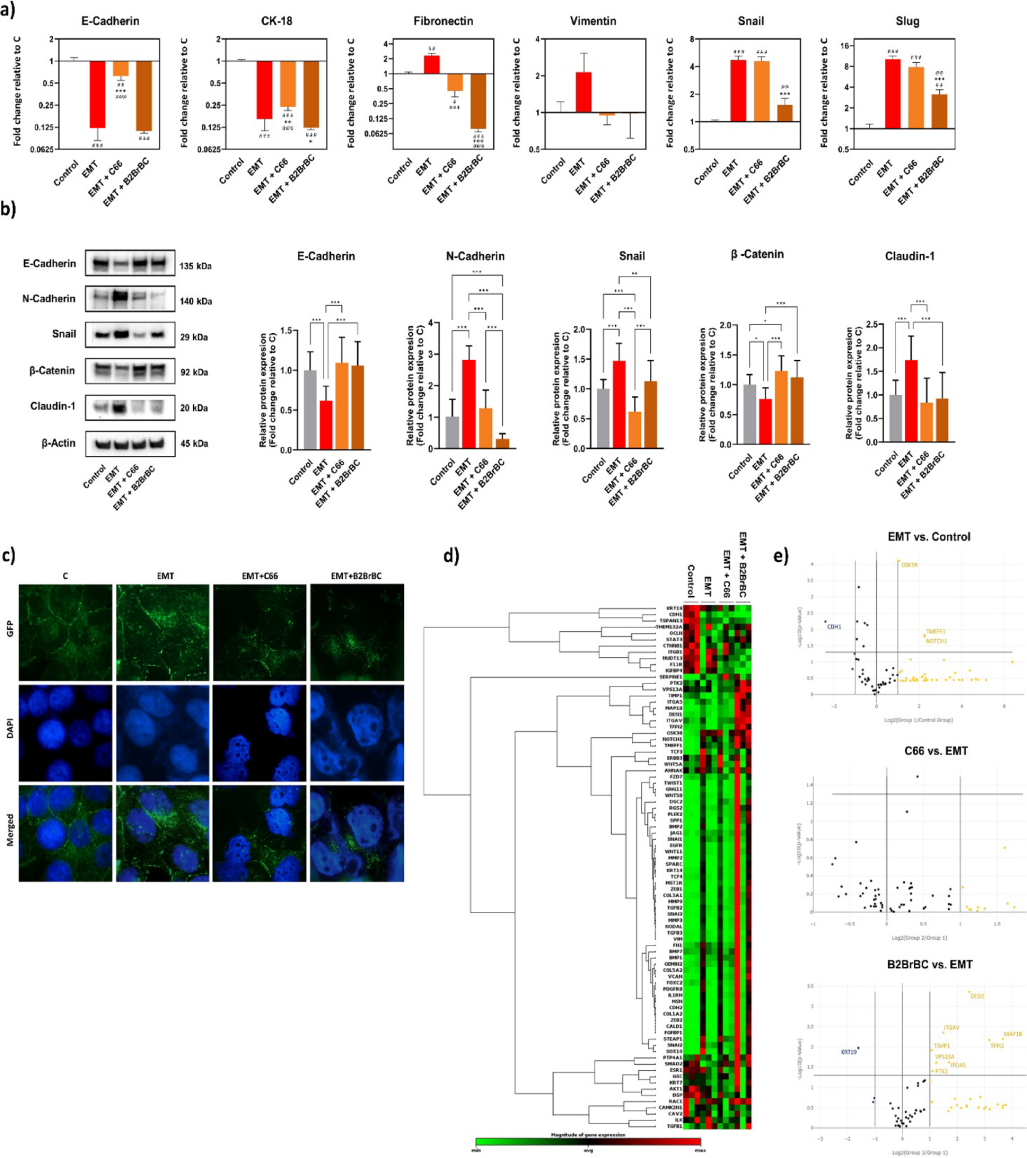
Effects of C66 and B2BrBC on EMT in MCF-7 breast cancer cells. Initially, cells were grown in a medium supplemented with an EMT-inducing cocktail for 48 h before C66 or B2BrBC (100 µM) was added for an additional 72 h. (**a**) qRT-PCR analysis was performed to measure mRNA expression of epithelial (*E-cadherin* and *CK-18*) and mesenchymal (*fibronectin*, *vimentin*, *snail*, and *slug*) markers. Data were calculated using the ΔΔ*Ct* method and are presented as fold-change (EMT, C66, or B2BrBC treatment), compared to control ± SEM. ^#^, compared to control; *, compared to EMT alone group; ^@^, C66 vs. B2BrBC; one symbol, *p* < 0.05; two symbols, *p* < 0.01; three symbols, *p* < 0.001. (**b**) Western blot and densitometry analyses of MCF-7 cells. Protein levels of E-cadherin, N-cadherin, snail, β-Catenin, and claudin-1 were evaluated, and β-Actin was used as a loading control. Data was presented as a relative protein expression (fold-change) of treatment vs. control ± SD. *, *p* < 0.05; **, *p* < 0.01; ***, *p* < 0.001. (**c**) Immunofluorescence staining for β-Catenin protein (green) of MCF-7 cells. Nuclei were counterstained with DAPI (blue). Magnification 20×. (**d**) Results from qRT-PCR array for genes linked to EMT in MCF-7 cells. Clustergram of the complete dataset presenting a heat map (displaying gene expression levels; red indicates high expression, green - low expression) with dendrograms (showing the clustering of samples and genes based on expression similarity across groups). **(e)** Volcano plots representing fold-change gene expression after treatment. Genes significantly upregulated or downregulated more than two-fold (*p* < 0.05) are listed in orange and blue, respectively

The results from RT-qPCR analyses demonstrated that treatment with the EMT cocktail decreased the mRNA expression of the epithelial (*E-cadherin* and *CK-18*) and increased that of the mesenchymal (*fibronectin*, *snail*, and *slug*) markers. The co-treatment with the EMT-inducer and C66 or B2BrBC reversed these trends, with the effect of C66 being more pronounced on the epithelial markers and B2BrBC on the mesenchymal markers (Fig. 5a). Similarly, Western blot analyses of E-cadherin, N-cadherin, and snail protein expression confirmed the RT-qPCR findings (Fig. 5b). The Densitometry analyses also showed that EMT induction in MCF-7 cells increased the protein expression of β-catenin while reducing the expression o*f* claudin-1. Co-treatment with the EMT-inducer and C66 or B2BrC was found to reverse these effects. Further, we performed immunofluorescence staining against β-catenin in MCF-7 cells subjected to the same treatment conditions. Following treatment with the EMT-inducer, the β-catenin exhibited more dispersed localization within the cells, compared to the primarily localized staining observed at the cell membranes in the control cells (Fig. 5c).

To investigate further the effect of C66 and B2BrBC on EMT, we performed an RT-qPCR array of 84 key genes involved in EMT using MCF-7 cells. The results revealed several notable changes in EMT-related gene expression after the EMT-inducing cocktail treatment (Fig. 5d and e). The heat map shows that among the most strongly upregulated genes in the EMT group were *SNAI1* (encoding for snail protein), *SNAI2* (slug), *TWIST1* (twist), and *VIM* (vimentin). The increased expression of these genes aligns with the morphological changes of EMT induction. Interestingly, co-treatment with the EMT-inducer and C66 or B2BrBC attenuated the EMT-induced upregulation of *SNAI1*, *SNAI2*, *TWIST1*, and *VIM*, suggesting that these compounds may inhibit EMT by modulating the expression of key transcriptional regulators. Additionally, the EMT-inducer treatment led to a downregulation of *CDH1* (E-cadherin) and *OCLN* (occludin), while the co-treatment with C66 or B2BrBC partially restored the expression of these epithelial markers, further supporting their role in inhibiting EMT. The EMT-inducing cocktail also upregulated *ZEB1* and *ZEB2*, but the co-treatment with C66 or B2BrBC attenuated their expression. Similarly, changes in the expression of genes involved in cell adhesion and extracellular matrix (ECM) remodeling, such as *MMP2*, *MMP9*, and *FN1* (fibronectin), followed the same trends, reflecting the impact on invasive and migratory properties of cells undergoing EMT.

### Comparative analysis of cancer metastasis markers expression in breast cancer cells treated with C66 and B2BrBC

To further investigate the effects of C66 and B2BrBC on the metastatic potential of breast cancer cells, we performed a MILLIPLEX array analysis measuring the expression levels of various proteins playing roles in cancer metastasis.

MCF-7 and BT-474 cells were treated with 100 µM of C66 or B2BrBC, and DMSO (vehicle control) for 24 h, and conditioned cell culture medium was subjected to analysis. The MILLIPLEX array included eight biomarkers with various roles in cancer, such as regulators of bone remodeling and mineralization (osteoprotegerin (OPG) and tartrate-resistant acid phosphatase 5 (TRAP5)), regulators of signaling pathways (Dickkopf-1 (DKK-1) and TNF-like weak inducer of apoptosis (TWEAK)), markers of cellular stress and inflammation (growth differentiation factor 15 (GDF15) and chitinase-3-like protein 1 (YKL40)), neuronal and neuroendocrine marker (neuron-specific enolase (NSE)), and matricellular protein involved in tissue remodeling (periostin).

**Fig. 6:**
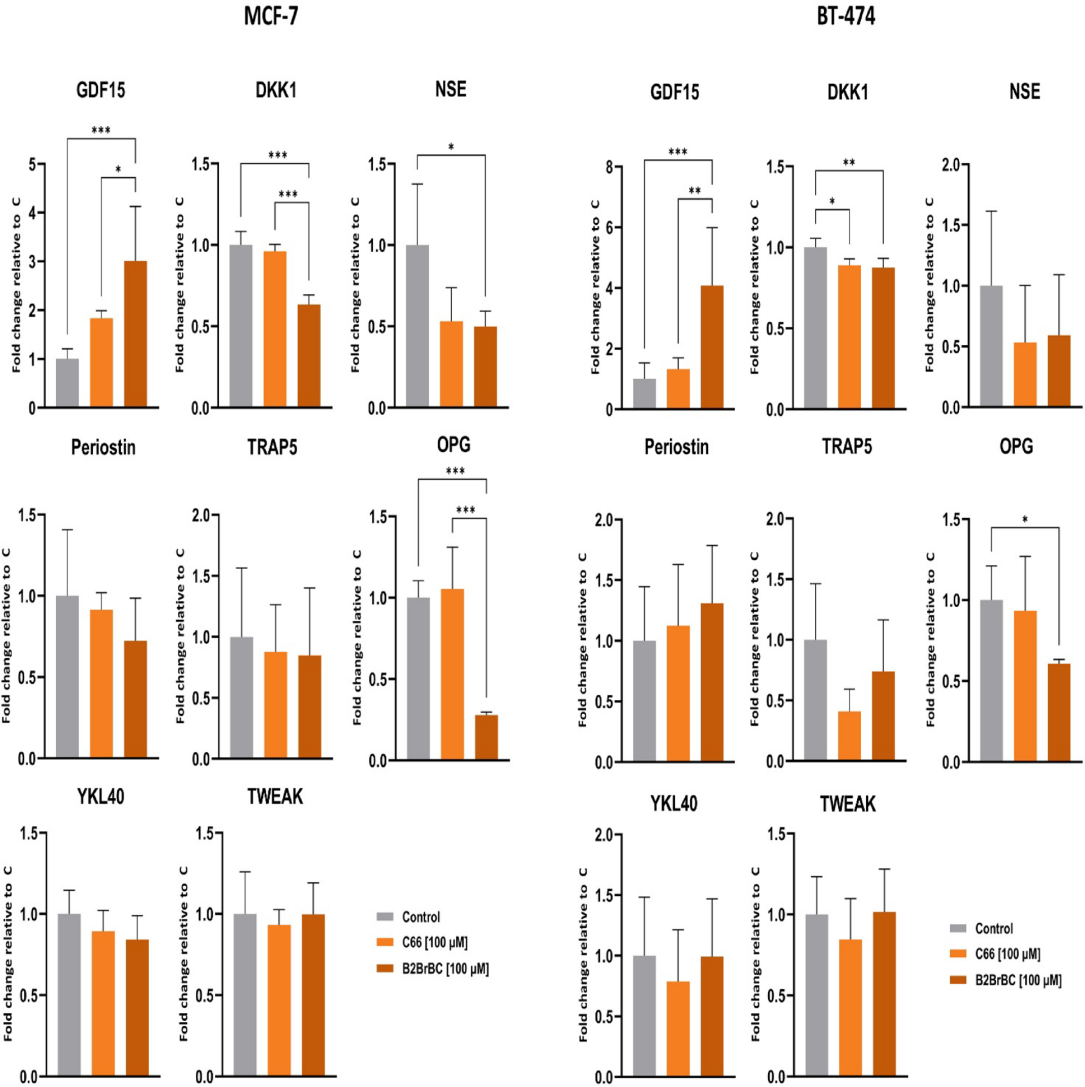
Effects of C66 and B2BrBC on cancer metastasis gene expression in MCF-7 and BT-474 breast cancer cells. MCF-7 and BT-474 breast cancer cells were subjected to C66 or B2BrBC treatment (100 µM) for 24 h, and a MILLIPLEX array analysis was performed to measure the expression levels of various proteins playing roles in cancer metastasis (OPG, TRAP5, DKK-1, TWEAK, GDF15, YKL40, NSE, and Periostin). The data were presented as a fold change compared to the control ± SD. *, *p* < 0.05; **, *p* < 0.01; ***, *p* < 0.001

The results from the analysis revealed differential expression of several biomarkers between the control, C66, and B2BrBC (Fig. 6). In the MCF-7 cells, DKK1, OPG, and NSE levels were decreased in cells treated with B2BrBC, compared to the control, while treatment with B2BrBC notably increased GDF15 levels. On the other hand, C66-treated cells did not significantly change the levels of any of the observed proteins. Similar trends were observed in the BT-474 cells, although the differences were less pronounced.

### In Silico evaluation of ADME properties of C66 and B2BrBC

Both compounds subjected to physicochemical properties prediction provided values that generally fall within the desired range, passed the examined filters for drug-likeness as good orally active drug candidates, and showed no more than one violation of the specified criteria (Table 1).

**Table 1:** Physicochemical properties representing the parameters of two drug-likeness filters, Lipinski and Veber

Compound	Molecular weight (g/mol)	Number of rotatable bonds	Number of H-bond acceptors	Number of H-bond donors	TPSA (Å²)	Log Po/w (MLOGP)	Lipinski	Veber	Bio- availability Score
Curcumin	368.38	5	6	3	96.22	1.47	Yes; 0 violation	Yes	0.56
C66	410.35	4	7	0	17.07	5.77	Yes; 1 violation: MLOGP > 4.15	Yes	0.55
B2BrBC	432.15	2	1	0	17.07	5.35	Yes; 1 violation: MLOGP > 4.15	Yes	0.55

There was one violation of Lipinski’s rule of five (MW ≤ 500; MlogP ≤ 4.15; N or O ≤ 10; NH or OH ≤ 5) regarding the MlogP values for the molecules (5.35 for B2BrBC and 5.77 for C66, respectively, due to the increased lipophilicity relative to CUR) and no violations of the Veber filter (rotatable bonds ≤ 10; TPSA ≤ 140). The assessed computational bioavailability score for the analogs gave favorable results and was comparable to that of CUR.

**Fig. 7:**
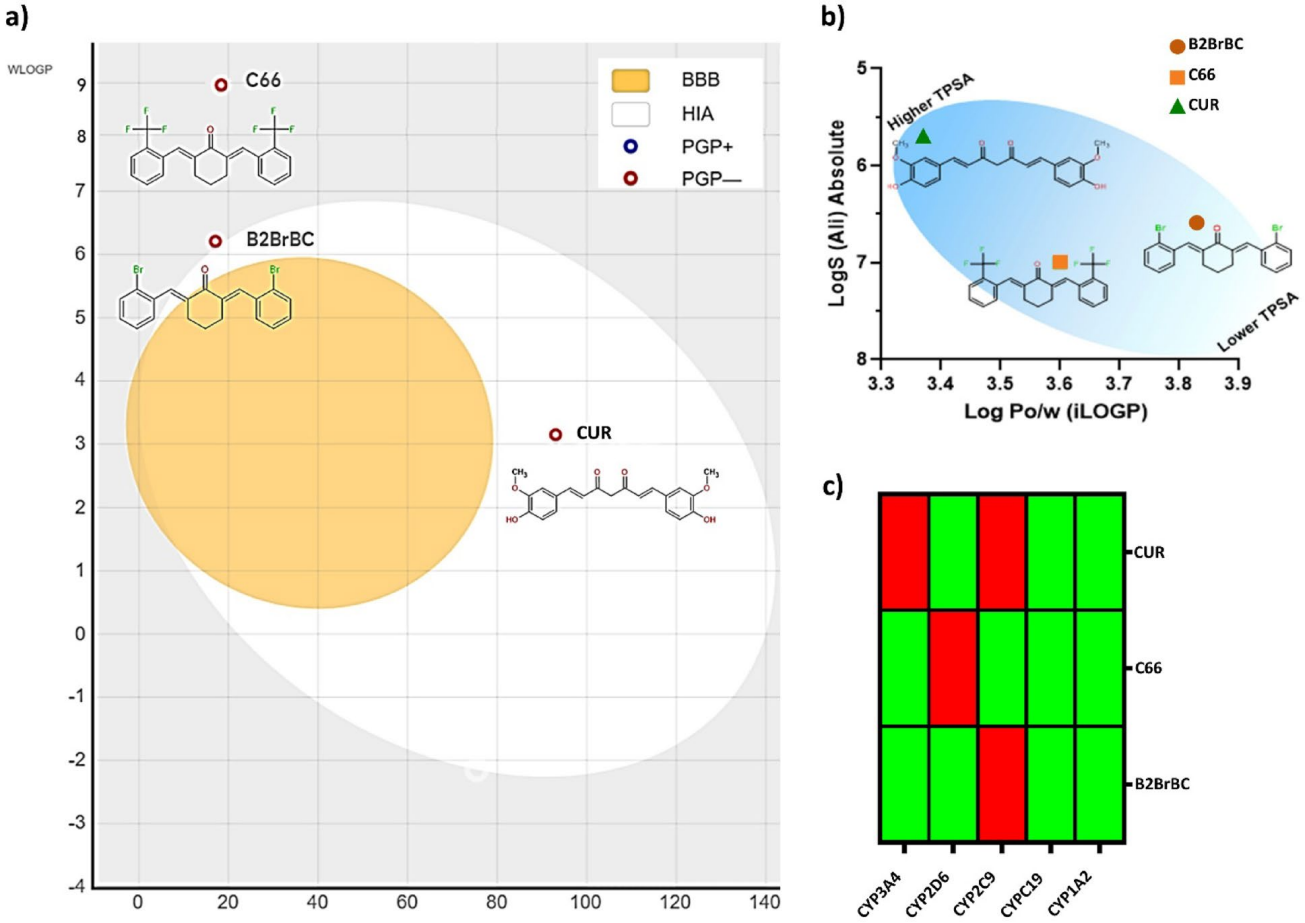
Absorption, distribution, metabolism, and excretion (ADME) properties, pharmacokinetic descriptors, and drug-likeness of C66, B2BrBC, and CUR. (**a**) Boiled egg model for prediction of GI absorption, BBB permeation, and P-gp substrate. The egg-shaped classification plot includes the yolk (i.e., the physicochemical area for highly probable BBB permeation) and the white (i.e., the physicochemical area for highly probable GI absorption). Blue points represent molecules predicted to be substrates of P-gp (PGP+), and red points are for non-substrate molecules of P-gp (PGP-). It can be observed that both analogs fall within the grey region and have red points. (**b**) Predicted values for solubility (y-axis: LogS (Ali)-absolute values), lipophilicity (x-axis: iLOGP), and polarity (Total polar surface area-blue region) of MACs. Note that both molecules were within the acceptable range of the log S scale: insoluble < − 10 < poorly < − 6 < moderately < − 4 < soluble < − 2 < very < 0 < highly; iLOGP ≤ 5; 20 Å^2^ < TPSA for normal polarity < 130 Å^2^ (marked with the blue ellipsoid-darker shades depict higher polarity). (**c**) Heat map depicting inhibition of five cytochrome P450 isoforms (x-axis) by the synthesized MACs (B2BrBC; C66; CUR). The inhibited P450 isoform is represented by a red bar, while the non-inhibited isoform by a green bar. The analogs showed less inhibition of the enzyme isoforms compared to CUR’s predicted effect

Passive intestinal (HIA) absorption and blood-brain barrier (BBB) permeation analysis, as predicted with the BOILED-Egg model, resulted in values within the grey region for both analogs, implying low predicted absorption and limited brain penetration (Fig. 7a). Furthermore, as illustrated in Fig. 7b, the predicted logS(Ali), iloqP, and TPSA values suggest poor water solubility for the analogs, compared to CUR’s moderate water solubility.

It can be noted that none of the MACs were substrates for the P-glycoprotein, which is important given the bypassed reduced drug bioavailability (Fig. 7a). Moreover, the predictions suggest that the analogs generally do not inhibit the five major isoforms of cytochromes P450 (CYP1A2, CYP2C19, CYP2C9, CYP2D6, CYP3A4) (Fig. 7c).

## Discussion

This study evaluated the stability, antioxidant capacity, and effects on viability, motility, and EMT in breast cancer cells of two MACs, C66 and B2BrBC. We hypothesized that structural modifications of CUR, such as removing the β-diketone moiety, adding a cyclohexanone ring, and incorporating distinct substituents on the phenyl rings (trifluoromethyl in C66 and bromine in B2BrBC), could improve stability and cellular target interactions, potentially enhancing therapeutic efficacy through modified pharmacological profiles.

Our data demonstrated that both C66 and B2BrBC exhibited significantly improved stability in solution at physiological pH compared to CUR, aligning with prior reports that β-diketone removal enhances pharmacokinetic profiles [22, 30]. In line with this, our hemolytic assay data suggested that these modifications in the tested MACs lead to similar hemolytic activity compared to CUR, demonstrating an equivalent safety profile.

The DPPH assay results indicated that C66 and B2BrBC exhibit negligible free-radical scavenging activity compared to CUR, with additional reductions in CAT and SOD activity in breast cancer cell lines. While this is in contrast with CUR’s antioxidant profile, reduced antioxidant capacity in cancer cells could be advantageous, enhancing their susceptibility to apoptosis, as supported by studies on structurally similar MACs [31–35]. It is important to note that the DPPH assay is just one of several methods to assess antioxidant capacity and may not fully capture these compounds’ antioxidant properties in biological systems. Additionally, in diabetic rat models, C66 and B2BrBC improved oxidative stress markers in various tissues [36], indicating context-dependent effects.

Our results from cytotoxicity assays demonstrated that both C66 and B2BrBC selectively reduced the viability of breast cancer cells while sparing MCF-10 A cells, supporting their safety in non-cancerous breast epithelial cells. Caspase-3 activation, serving as a complementary assay to the MTT assay, strongly suggests apoptosis as a primary mechanism of cell death, resembling CUR’s tumor-suppressing effects through caspase cascades and NF-κB and JNK downregulation [37], although other processes like necrosis or cell cycle arrest cannot be fully excluded. Previous studies have shown that various CUR analogs inhibit the NF-κB pathway in lung, liver, breast, gastric, thyroid, pancreatic cancer, and cholangiocarcinoma cells [38–41], promoting apoptosis. Cell line-specific patterns of caspase-3 activation, i.e., B2BrBC in MCF-7 and MDA-MB-231 cells or C66 in BT-474 cells, imply that structural differences between C66 and B2BrBC influence their respective apoptotic pathways. Both C66 and B2BrBC are known JNK inhibitors [36, 42], and their cytotoxicity may involve disrupting stress response pathways in cancer cells. Future studies should confirm whether JNK and NF-κB inhibition are central to the anticancer activities of these compounds.

Wound-healing and Transwell migration assays revealed potent antimigratory actions, with B2BrBC outperforming C66, likely due to its steric and electronic properties. This migrastatic potential, also seen in other MACs like PGV-1 and CCA-1.1, underscores their ability to suppress EMT [43, 44]. RT-qPCR data and Western blot analyses confirmed that C66 and B2BrBC reversed EMT-inducer effects, upregulating epithelial markers (*CDH1*, *OCLN*) and downregulating mesenchymal markers (*SNAI1*, *SNAI2*,* VIM*). Immunofluorescence further showed reduced β-catenin’s nuclear translocation, suggesting interference with Wnt/β-catenin signaling, a known target of CUR derivatives, thus reducing the breast cancer cells’ invasive potential [45, 46].

MILLIPLEX array analysis demonstrated cell line-specific effects on metastasis-related proteins. B2BrBC downregulated DKK1 in MCF-7 and BT-474 cells, potentially reducing osteolytic breast cancer bone metastasis by promoting osteoblast activity [47], but upregulated GDF15 in MCF-7 cells, highlighting its dual role in metastasis depending on the cancer stage [48–51]. B2BrBC also downregulated OPG in MCF-7 cells, potentially lowering metastatic risk [52–54]. These differential effects emphasize the importance of considering the heterogeneity of breast cancer when evaluating the effects of these compounds [55]. However, the functional roles of these proteins in mediating the effects of C66 and B2BrBC remain speculative, and future studies should include siRNA knockdown or overexpression experiments to confirm their contributions to breast cancer progression.

Our in silico ADME analysis indicated that both C66 and B2BrBC met key pharmacokinetic criteria, adhering to Veber’s rule (molecular flexibility and polar surface area) and Lipinski’s rule of five (molecular weight, lipophilicity, and hydrogen bonding potential), which are linked to good oral absorption and drug-likeness. The compounds’ favorable bioavailability scores support the hypothesis that they could achieve improved systemic concentrations over CUR. However, both C66 and B2BrBC exhibited reduced solubility (bulky groups and heavy atoms), which may impact in vivo pharmacokinetics. The compounds’ ability to evade efflux by P-glycoprotein, as demonstrated in our analysis, may offset this drawback by allowing higher intracellular concentrations and reducing the risk of rapid excretion [56]. This characteristic enhances therapeutic efficacy, as P-glycoprotein often contributes to multidrug resistance in cancer therapy. Additionally, the unlikeliness of C66 and B2BrBC to inhibit the five major cytochrome P450 isoforms further suggests a low risk of pharmacokinetics-related drug-drug interactions [57]. These in silico predictions warrant in vivo validation of the clinical potential of these compounds.

Our study provides a foundation for understanding C66 and B2BrBC’s effects on breast cancer cells, highlighting their stability, selective cytotoxicity, and antimetastatic potential. However, the study’s limitations include the in vitro setting, which may not fully represent the complex interactions and microenvironment effects present within a living organism. Although C66 and B2BrBC showed promising cytotoxic and antimigratory effects against breast cancer cells, the detailed mechanisms underlying these effects remain unexplored. While our data strongly support the fact that C66 and B2BrBC attenuate EMT of breast cancer cells, further investigations are needed to clarify the precise mechanisms by which these two compounds regulate EMT-associated markers, including the involvement of other signaling pathways, like TGF-β, beyond the Wnt pathway we assessed via β-catenin. Additionally, functional assays, such as spheroid invasion assays, are needed to confirm phenotypic EMT reversal. While we assessed stability in simulated physiological conditions, detailed pharmacokinetic analyses, such as plasma stability and half-life measurements, are needed to further validate their clinical potential. Future studies should explore the potential synergistic effects of these compounds with conventional chemotherapeutic agents. Lastly, i*n vivo* studies would allow a more comprehensive comparison with curcumin, highlighting the therapeutic advantages of C66 and B2BrBC due to their improved bioavailability and stability.

## Conclusion

Our study demonstrates that the structural modifications in C66 and B2BrBC result in enhanced stability and selective cytotoxicity against breast cancer cells. The decreased solubility of these compounds is offset by their improved stability and other positive predicted pharmacokinetic properties. Although their free-radical scavenging capacity is negligible compared to curcumin, their potential to selectively induce apoptosis renders them promising candidates for further development. Additionally, their ability to inhibit EMT and cell migration highlights their potential in antimetastatic therapies. Further studies are required to investigate the molecular mechanisms underlying their anticancer effects, particularly in relation to JNK and NF-kB signaling pathways.

## Supplementary Information

Supplementary Material 1

## Data Availability

Data is provided within the manuscript or supplementary information files.

## Abbreviations

ADME: Absorption, distribution, metabolism, and excretion
B2BrBC: (2E,6E)-2,6-bis(2-bromobenzylidene)cyclohexanone
C66: (2E,6E)-2,6-bis[(2-trifluoromethyl)benzylidene]cyclohexanone
CAT: Catalase
CDH1: Cadherin 1 (E-Cadherin)
DMSO: Dimethyl sulfoxide
DKK1: Dickkopf WNT signaling pathway inhibitor 1
DPPH: 2,2-Diphenyl-1-picrylhydrazyl
EC_50_: Half-maximal effective concentration
EMT: Epithelial-to-mesenchymal transition
FBS: Fetal bovine serum
GDF15: Growth differentiation factor 15
HIA: Human intestinal absorption
JNK: c-Jun N-terminal Kinase
MACs: Monocarbonyl analogs of curcumin
NF-κB: Nuclear factor kappa-light-chain-enhancer of activated B cells
NSE: Neuron-specific enolase
OCLN: Occludin
OPG: Osteoprotegerin
PBS: Phosphate-buffered saline
RBCs: Red blood cells
RIPA: Radioimmunoprecipitation assay
RSA%: Radical scavenging activity percentage
RT-qPCR: Reverse transcription quantitative polymerase chain reaction
SNAI1: Snail family transcriptional repressor 1
SNAI2: Snail family transcriptional repressor 2
SOD: Superoxide dismutase
SDS-PAGE: Sodium dodecyl sulfate-polyacrylamide gel electrophoresis
TGFβ: Transforming growth factor beta
TRAP5: Tartrate-resistant acid phosphatase 5
TWEAK: TNF-like weak inducer of apoptosis
TWIST1: Twist family BHLH transcription factor 1
VIM: Vimentin
ZEB1: Zinc finger E-box binding homeobox 1
ZEB2: Zinc finger E-box binding homeobox 2
