# Supplementary material for: Assessment of the anticancer and antimetastatic effects of monocarbonyl analogs of curcumin, C66 and B2BrBC, in breast cancer cells
Source: Cancer Cell Int. 2026 Jan 21;26:90. doi: 10.1186/s12935-026-04184-8 (PMC12905848; doi:10.1186/s12935-026-04184-8)
Supplement: Supplementary file 1 — Supplementary Material 1 [file 12935_2026_4184_MOESM1_ESM.docx]

**Supplementary Table 1. List of primers used for the qRT-PCR analyses.**

| **Gene name** | **Species** | **Forward/Reverse primer (5’-3’)** | **Primer length [bp]** | **GC%** | **Predicted Tm [°C]** | **Source** |
| --- | --- | --- | --- | --- | --- | --- |
| *CDH1* | Hu | TGCCCAGAAAATGAAAAAGG  GTGTATGTGGCAATGCGTTC | 20  20 | 40  50 | 54.62  58.10 | [24] |
| *KRT18* | Hu | CCAGTCTGTGGAGAACGACA CTGAGATTTGGGGGCATCTA | 20  20 | 55  50 | 59.33  56.67 | [24] |
| *FN1* | Hu | ATGATGAGGTGCACGTGTGT  CTCTGAATCCTGGCATTGGT | 20  20 | 50  50 | 59.96  59.10 | [24] |
| *VIM* | Hu | AGATGGCCCTTGACATTGAG  TGGAAGAGGCAGAGAAATCC | 20  20 | 50  50 | 57.57  56.91 | [24] |
| *SNAI1*  (*s*. Snail) | Hu | AAGATGCACATCCGAAGCCA  CAAAAACCCACGCAGACAGG | 20  20 | 50  55 | 60.04  59.97 | [25] |
| *SNAI2*  (*s*. Slug) | Hu | CTTCCTGGTCAAGAAGCA  GGGAAATAATCACTGTATGTGTG | 18  23 | 55  45 | 54.06  55.38 | [26] |
| *GAPDH* | Hu, Mu, | CCTGCACCACCAACTGCTTA | 20 | 55 | 60.54 | [27] |
|  | Rt | GGCCATCCACAGTCTTCTGAG | 21 | 57 | 60.41 |  |

**Supplementary Table 2. List of genes included in the PCR array.**

| **Gene Symbol** | **Gene full name** | **UniGene ID** | **GenBank ID** |
| --- | --- | --- | --- |
| AHNAK | AHNAK nucleoprotein | Hs.502756 | NM_024060 |
| AKT1 | V-akt murine thymoma viral oncogene homolog 1 | Hs.525622 | NM_005163 |
| BMP1 | Bone morphogenetic protein 1 | Hs.1274 | NM_006129 |
| BMP2 | Bone morphogenetic protein 2 | Hs.73853 | NM_001200 |
| BMP7 | Bone morphogenetic protein 7 | Hs.473163 | NM_001719 |
| CALD1 | Caldesmon 1 | Hs.490203 | NM_004342 |
| CAMK2N1 | Calcium/calmodulin-dependent protein kinase II inhibitor 1 | Hs.197922 | NM_018584 |
| CAV2 | Caveolin 2 | Hs.212332 | NM_001233 |
| CDH1 | Cadherin 1, type 1, E-cadherin (epithelial) | Hs.461086 | NM_004360 |
| CDH2 | Cadherin 2, type 1, N-cadherin (neuronal) | Hs.464829 | NM_001792 |
| COL1A2 | Collagen, type I, alpha 2 | Hs.489142 | NM_000089 |
| COL3A1 | Collagen, type III, alpha 1 | Hs.443625 | NM_000090 |
| COL5A2 | Collagen, type V, alpha 2 | Hs.445827 | NM_000393 |
| CTNNB1 | Catenin (cadherin-associated protein), beta 1, 88kDa | Hs.476018 | NM_001904 |
| DSC2 | Desmocollin 2 | Hs.95612 | NM_004949 |
| DSP | Desmoplakin | Hs.519873 | NM_004415 |
| EGFR | Epidermal growth factor receptor | Hs.488293 | NM_005228 |
| ERBB3 | V-erb-b2 erythroblastic leukemia viral oncogene homolog 3 (avian) | Hs.118681 | NM_001982 |
| ESR1 | Estrogen receptor 1 | Hs.208124 | NM_000125 |
| F11R | F11 receptor | Hs.517293 | NM_016946 |
| FGFBP1 | Fibroblast growth factor binding protein 1 | Hs.1690 | NM_005130 |
| FN1 | Fibronectin 1 | Hs.203717 | NM_002026 |
| FOXC2 | Forkhead box C2 (MFH-1, mesenchyme forkhead 1) | Hs.436448 | NM_005251 |
| FZD7 | Frizzled family receptor 7 | Hs.173859 | NM_003507 |
| GNG11 | Guanine nucleotide binding protein (G protein), gamma 11 | Hs.83381 | NM_004126 |
| GSC | Goosecoid homeobox | Hs.440438 | NM_173849 |
| GSK3B | Glycogen synthase kinase 3 beta | Hs.445733 | NM_002093 |
| IGFBP4 | Insulin-like growth factor binding protein 4 | Hs.462998 | NM_001552 |
| IL1RN | Interleukin 1 receptor antagonist | Hs.81134 | NM_000577 |
| ILK | Integrin-linked kinase | Hs.5158 | NM_004517 |
| ITGA5 | Integrin, alpha 5 (fibronectin receptor, alpha polypeptide) | Hs.505654 | NM_002205 |
| ITGAV | Integrin, alpha V (vitronectin receptor, alpha polypeptide, antigen CD51) | Hs.436873 | NM_002210 |
| ITGB1 | Integrin, beta 1 (fibronectin receptor, beta polypeptide, antigen CD29 includes  MDF2, MSK12) | Hs.643813 | NM_002211 |
| JAG1 | Jagged 1 | Hs.728907 | NM_000214 |
| KRT14 | Keratin 14 | Hs.654380 | NM_000526 |
| KRT19 | Keratin 19 | Hs.654568 | NM_002276 |
| KRT7 | Keratin 7 | Hs.411501 | NM_005556 |
| MAP1B | Microtubule-associated protein 1B | Hs.335079 | NM_005909 |
| MMP2 | Matrix metallopeptidase 2 (gelatinase A, 72kDa gelatinase, 72kDa type IV collagenase) | Hs.513617 | NM_004530 |
| MMP3 | Matrix metallopeptidase 3 (stromelysin 1, progelatinase) | Hs.375129 | NM_002422 |
| MMP9 | Matrix metallopeptidase 9 (gelatinase B, 92kDa gelatinase, 92kDa type IV collagenase) | Hs.297413 | NM_004994 |
| MSN | Moesin | Hs.87752 | NM_002444 |
| MST1R | Macrophage stimulating 1 receptor (c-met-related tyrosine kinase) | Hs.517973 | NM_002447 |
| NODAL | Nodal homolog (mouse) | Hs.370414 | NM_018055 |
| NOTCH1 | Notch 1 | Hs.495473 | NM_017617 |
| NUDT13 | Nudix (nucleoside diphosphate linked moiety X)-type motif 13 | Hs.533657 | NM_015901 |
| OCLN | Occludin | Hs.592605 | NM_002538 |
| PDGFRB | Platelet-derived growth factor receptor, beta polypeptide | Hs.509067 | NM_002609 |
| PLEK2 | Pleckstrin 2 | Hs.170473 | NM_016445 |
| PPPDE2 | PPPDE peptidase domain containing 2 | Hs.570455 | NM_015704 |
| PTK2 | PTK2 protein tyrosine kinase 2 | Hs.395482 | NM_005607 |
| PTP4A1 | Protein tyrosine phosphatase type IVA, member 1 | Hs.227777 | NM_003463 |
| RAC1 | Ras-related C3 botulinum toxin substrate 1 (rho family, small GTP binding protein Rac1) | Hs.413812 | NM_006908 |
| RGS2 | Regulator of G-protein signaling 2, 24kDa | Hs.78944 | NM_002923 |
| SERPINE1 | Serpin peptidase inhibitor, clade E (nexin, plasminogen activator inhibitor type 1), member 1 | Hs.414795 | NM_000602 |
| SIP1 | Survival of motor neuron protein interacting protein 1 | Hs.708127 | NM_003616 |
| SMAD2 | SMAD family member 2 | Hs.12253 | NM_005901 |
| SNAI1 | Snail homolog 1 (Drosophila) | Hs.48029 | NM_005985 |
| SNAI2 | Snail homolog 2 (Drosophila) | Hs.360174 | NM_003068 |
| SNAI3 | Snail homolog 3 (Drosophila) | Hs.253790 | NM_178310 |
| SOX10 | SRY (sex determining region Y)-box 10 | Hs.376984 | NM_006941 |
| SPARC | Secreted protein, acidic, cysteine-rich (osteonectin) | Hs.111779 | NM_003118 |
| SPP1 | Secreted phosphoprotein 1 | Hs.313 | NM_000582 |
| STAT3 | Signal transducer and activator of transcription 3 (acute-phase response factor) | Hs.463059 | NM_003150 |
| STEAP1 | Six transmembrane epithelial antigen of the prostate 1 | Hs.61635 | NM_012449 |
| TCF3 | Transcription factor 3 (E2A immunoglobulin enhancer binding factors E12/E47) | Hs.371282 | NM_003200 |
| TCF4 | Transcription factor 4 | Hs.644653 | NM_003199 |
| TFPI2 | Tissue factor pathway inhibitor 2 | Hs.438231 | NM_006528 |
| TGFB1 | Transforming growth factor, beta 1 | Hs.645227 | NM_000660 |
| TGFB2 | Transforming growth factor, beta 2 | Hs.133379 | NM_003238 |
| TGFB3 | Transforming growth factor, beta 3 | Hs.592317 | NM_003239 |
| TIMP1 | TIMP metallopeptidase inhibitor 1 | Hs.522632 | NM_003254 |
| TMEFF1 | Transmembrane protein with EGF-like and two follistatin-like domains 1 | Hs.598100 | NM_003692 |
| TMEM132A | Transmembrane protein 132A | Hs.118552 | NM_178031 |
| TSPAN13 | Tetraspanin 13 | Hs.364544 | NM_014399 |
| TWIST1 | Twist homolog 1 (Drosophila) | Hs.66744 | NM_000474 |
| VCAN | Versican | Hs.643801 | NM_004385 |
| VIM | Vimentin | Hs.642813 | NM_003380 |
| VPS13A | Vacuolar protein sorting 13 homolog A (S. cerevisiae) | Hs.459790 | NM_033305 |
| WNT11 | Wingless-type MMTV integration site family, member 11 | Hs.108219 | NM_004626 |
| WNT5A | Wingless-type MMTV integration site family, member 5A | Hs.696364 | NM_003392 |
| WNT5B | Wingless-type MMTV integration site family, member 5B | Hs.306051 | NM_032642 |
| ZEB1 | Zinc finger E-box binding homeobox 1 | Hs.124503 | NM_030751 |
| ZEB2 | Zinc finger E-box binding homeobox 2 | Hs.34871 | NM_014795 |
| ACTB | Actin, beta | Hs.520640 | NM_001101 |
| B2M | Beta-2-microglobulin | Hs.534255 | NM_004048 |
| GAPDH | Glyceraldehyde-3-phosphate dehydrogenase | Hs.592355 | NM_002046 |
| HPRT1 | Hypoxanthine phosphoribosyltransferase 1 | Hs.412707 | NM_000194 |
| RPLP0 | Ribosomal protein, large, P0 | Hs.546285 | NM_001002 |
| HGDC | Human Genomic DNA Contamination | N/A | SA_00105 |
| RTC | Reverse Transcription Control | N/A | SA_00104 |
| RTC | Reverse Transcription Control | N/A | SA_00104 |
| RTC | Reverse Transcription Control | N/A | SA_00104 |
| PPC | Positive PCR Control | N/A | SA_00103 |
| PPC | Positive PCR Control | N/A | SA_00103 |
| PPC | Positive PCR Control | N/A | SA_00103 |

**Supplementary Table 3.** **List of genes in the PCR array organized by function.**

| **Function** | **Gene** |
| --- | --- |
| Up-Regulated During Epithelial-to-Mesenchymal Transition | AHNAK, BMP1, CALD1, CAMK2N1, CDH2 (N-Cadherin), COL1A2, COL3A1, COL5A2, FN1, FOXC2, GNG11, GSC, IGFBP4, ITGA5, ITGAV, MMP2, MMP3, MMP9, MSN, SERPINE1 (PAI-1), SNAI1 (SNAIL), SNAI2, SNAI3, SOX10, SPARC, STEAP1, TCF4, TIMP1, TMEFF1, TMEM132A, TWIST1, VCAN, VIM, VPS13A, WNT5A, WNT5B. |
| Down-Regulated During Epithelial-to-Mesenchymal Transition | CAV2, CDH1 (E-Cadherin), DESI1, DSP, FGFBP1, IL1RN, KRT19, MST1R (RON), NUDT13, OCLN, RGS2, SPP1, TFPI2, TSPAN13. |
| Differentiation & Development | AKT1, BMP1, BMP2, BMP7, COL3A1, COL5A2, CTNNB1, DSP, ERBB3, F11R, FOXC2, FZD7, GSC, JAG1, KRT14, MST1R(RON), NODAL,NOTCH1,PTP4A1,SMAD2 (MADH2),SNAI1 (SNAIL), SNAI2, SOX10, TGFB2, TGFB3, TMEFF1, TWIST1, VCAN, WNT11, WNT5A, WNT5B. |
| Cell Morphogenesis | CTNNB1, FOXC2, JAG1, RAC1, SMAD2 (MADH2), SNAI1 (SNAIL), SOX10, TGFB1, TGFB2, TGFB3, TWIST1, WNT11, WNT5A. |
| Cell Growth & Proliferation | AKT1, BMP1, BMP7, CAV2, CTNNB1, EGFR (ERBB1), ERBB3, FGFBP1, FOXC2, IGFBP4, ILK, JAG1, MST1R (RON), NODAL, PDGFRB, TGFB1, TGFB2, TGFB3, TIMP1, VCAN, ZEB1. |
| Cell Migration & Motility | CALD1, CAV2, EGFR (ERBB1), FN1, ITGB1, JAG1, MSN, MST1R (RON), NODAL, PDGFRB, RAC1, STAT3, TGFB1, VIM. |
| Cytoskeleton Regulators | CAV2, KRT7, MAP1B, PLEK2, RAC1, VIM. |
| Extracellular Matrix (ECM) & Cell Adhesion Molecules | BMP1, BMP7, CDH1 (E-Cadherin), CDH2 (N-Cadherin), COL1A2, COL3A1, COL5A2, CTNNB1, DSC2, EGFR (ERBB1), ERBB3, F11R, FN1, FOXC2, ILK, ITGA5, ITGAV, ITGB1, MMP2, MMP3, MMP9, PTK2 (FAK), RAC1, SERPINE1 (PAI-1), SPP1, TGFB1, TGFB2, TIMP1, VCAN. |
| Signal Transduction |  |
| Estrogen Receptor Signaling | CAV2, ESR1 (ERα), KRT19, TGFB3. |
| G-Protein Coupled Receptor Signaling | AKT1, FZD7, GNG11, RAC1, RGS2. |
| Integrin-Mediated Signaling | COL3A1, ILK, ITGA5, ITGAV, ITGB1, PTK2 (FAK). |
| Notch Signaling  Receptor Tyrosine Kinase Signaling  TGFβ / BMP Signaling  WNT Signaling  Transcription Factors | FOXC2, JAG1, NOTCH1.  EGFR (ERBB1), ERBB3, PDGFRB, RGS2, SPARC.  BMP1, BMP2, BMP7, COL3A1, SMAD2 (MADH2), TGFB1, TGFB2, TGFB3.  CTNNB1, FZD7, GSK3B, WNT11, WNT5A, WNT5B.  CTNNB1, ESR1 (ERα), FOXC2, GEMIN2, GSC, NOTCH1, SMAD2 (MADH2), SNAI2, SNAI3, SOX10, STAT3, TCF3, TCF4, TWIST1, ZEB1, ZEB2. |

**Supplementary Table 4: List of primary and secondary antibodies used for the Western blot analyses.**

| **Antibody** | **Species** | **Catalog #** | **Company** | **Dilution** |
| --- | --- | --- | --- | --- |
| E-Cadherin | Rabbit | 3195 | Cell Signaling Technology | 1:1000 |
| N-Cadherin | Rabbit | 13116 | Cell Signaling Technology | 1:1000 |
| Snail | Rabbit | 3879 | Cell Signaling Technology | 1:1000 |
| β -Catenin | Rabbit | 8480 | Cell Signaling Technology | 1:1000 |
| Claudin-1 | Rabbit | 13255 | Cell Signaling Technology | 1:1000 |
| β-Actin | Mouse | 3700 | Cell Signaling Technology | 1:1000 |
| anti-Rb IgG | Goat | 31460 | Thermo Fisher Scientific | 1:5000 |
| anti-Mo IgG | Goat | 31430 | Thermo Fisher Scientific | 1:5000 |

**Supplementary Table 5: Concentration at which 50% DPPH scavenging activity was achieved for the tested compounds.**

| **Compound** | **EC_50_** |
| --- | --- |
| Ascorbic acid | 49.2 μM (46.6 - 52.2, 95% CI) |
| Curcumin | 84.7 μM (78.7 - 91.8, 95% CI) |
| C66 | No detectable scavenging activity up to 4837 μM (2000 μg/ml) |
| B2BrBC | No detectable scavenging activity up to 4629 μM (2000 μg/ml) |
